# Effect of acute exercise on RBC deformability and RBC nitric oxide synthase signalling pathway in young sickle cell anaemia patients

**DOI:** 10.1038/s41598-019-48364-1

**Published:** 2019-08-14

**Authors:** Marijke Grau, Max Jerke, Elie Nader, Alexander Schenk, Celine Renoux, Bianca Collins, Thomas Dietz, Daniel Alexander Bizjak, Philippe Joly, Wilhelm Bloch, Philippe Connes, Aram Prokop

**Affiliations:** 1 German Sport University Cologne, Department of Molecular and Cellular Sports Medicine, Cologne, Germany; 2 University of Lyon, University Claude Bernard Lyon 1, Interuniversity Laboratory of Human Movement Biology EA7424, “Vascular Biology and Red Blood Cell” team, Villeurbanne, France; 3 Laboratory of Excellence “GR-Ex”, Paris, France; 4 East Biology Centre, UF “Biochemistry of Red Blood Cell Disease”, Academic Hospital of Lyon, HCL, Lyon, France; 5 Children’s Hospital Amsterdamer Straße Cologne; Clinic for Children and Youth Medicine, Paediatric Oncology/Haematology, Cologne, Germany

**Keywords:** Sickle cell disease, Diagnostic markers

## Abstract

Sickle cell anaemia (SCA) is characterized by reduced red blood cell (RBC) deformability and nitric oxide (NO) bioavailability. The aim of the study was to investigate whether exercise might affect these parameters in SCA. SCA patients and healthy controls (AA) performed an acute submaximal exercise test until subjects reached the first ventilatory threshold (VT 1). Blood was sampled at rest and at VT 1. At rest, free haemoglobin level was higher and RBC count, haemoglobin and haematocrit were lower in SCA compared to AA. RBC deformability was lower in SCA. Exercise had no effect on the tested parameters. RBC NO level was higher in SCA compared to AA at rest and significantly decreased after exercise in SCA. This might be related to a reduction in RBC-NO synthase (RBC-NOS) activation which was only observed in SCA after exercise. Free radical levels were higher in SCA at rest but concentration was not affected by exercise. Marker for lipid peroxidation and antioxidative capacity were similar in SCA and AA and not affected by exercise. In conclusion, a single acute submaximal bout of exercise has no deleterious effects on RBC deformability or oxidative stress markers in SCA, and seems to modulate RBC-NOS signalling pathway.

## Introduction

Sickle cell disease is the most frequent genetic disease in the world, with sickle cell anaemia (SCA; HbSS) reaching the highest prevalence. The gene defect of SCA is a single nucleotide mutation (adenine → thymine) in the β-globin gene, which results in the substitution of valine for glutamic acid in the sixth position of the beta chain of the haemoglobin S (HbS). HbS molecules aggregate and form fibrous precipitates under deoxygenated conditions. Such a phenomenon is called “HbS polymerization” and causes the sickling of red blood cells (RBC). Sickle RBC are very rigid and fragile, which may explain why SCA patients are prone to vaso-occlusive crises and are characterized by the presence of severe haemolytic anaemia^1^. SCA has been classified as a haemorheological disease and the severe reduction of RBC deformability is considered to be one of the primary factors responsible for the vaso-occlusive events and progressive organ damages in these patients^2–5^.

RBC need to be highly deformable to flow easily through the microcirculation and supply oxygen to the tissues and organs. Several factors determine the ability of RBC to deform such as the membrane deformability/elasticity, internal viscosity and cell sphericity (i.e., the surface-volume ratio)^6,7^. Recently, RBC nitric oxide (NO) content has been shown to modulate deformability of RBC^8–10^. NO production in RBC has been associated to RBC-NO synthase (RBC-NOS) activation. Although functionality of RBC-NOS was doubted earlier^11^, majority of published papers agree regarding the functional activity of RBC-NOS^8,9,12–14^. Thus, activation of Akt kinase leads to RBC-NOS activation which is reflected by phosphorylation of the RBC-NOS serine 1177 residue^8,9^. In contrast, phosphorylation of RBC-NOS residues serine 114 and threonine 495 have been associated to reduced RBC-NOS activation and NO production, respectively^15,16^. In healthy subjects, it has been suggested that RBC-NOS produced NO leads to S-nitrosylation of the cytoskeletal proteins α- and β spectrin, which in turn affects RBC deformability^9^. SCA patients show reduced RBC deformability but higher RBC-NOS dependent NO production and higher S-nitrosylation of the spectrins compared to healthy subjects suggesting that this higher NO production might not sufficiently affect RBC deformability to at least in part counterbalance the adverse effects of increased free radical levels on RBC integrity in SCA^17,18^.

Although controversies have been reported^19,20^, acute exercise has been shown to improve RBC deformability, depending on the population tested, volume and intensity of exercise^21–24^. One of the mechanisms suspected to be at the origin of the RBC deformability improvement in healthy individuals is the greater RBC-NOS activation and RBC NO production noted in response to exercise^16^. In addition, long term training is associated to a reduction in free radical production and improved antioxidant capacity in healthy people and subjects with metabolic diseases^25,26^. In SCA, the metabolic changes that occur during acute exercise, including increased free radical production, can promote HbS polymerization and RBC sickling. Thus, physicians and researchers do not really know what SCA patients are capable of (in term of exercise intensity and duration) without any risk to develop adverse clinical events^27^. It has been suggested that SCA individuals may undertake mild to moderate physical activities without any risk of acute clinical complications^28–30^. However, no study investigated the effects of acute exercise on RBC physiology and rheology in SCA. The aim of the present study was to investigate the effect of an acute submaximal exercise bout on RBC physiology, including RBC deformability, RBC NO signalling parameters and oxidative stress markers in SCA patients, to estimate whether exercise might be a possible risk or beneficial for RBC function.

## Results

### Physical performance is lower in SCA

**Figure 1:**
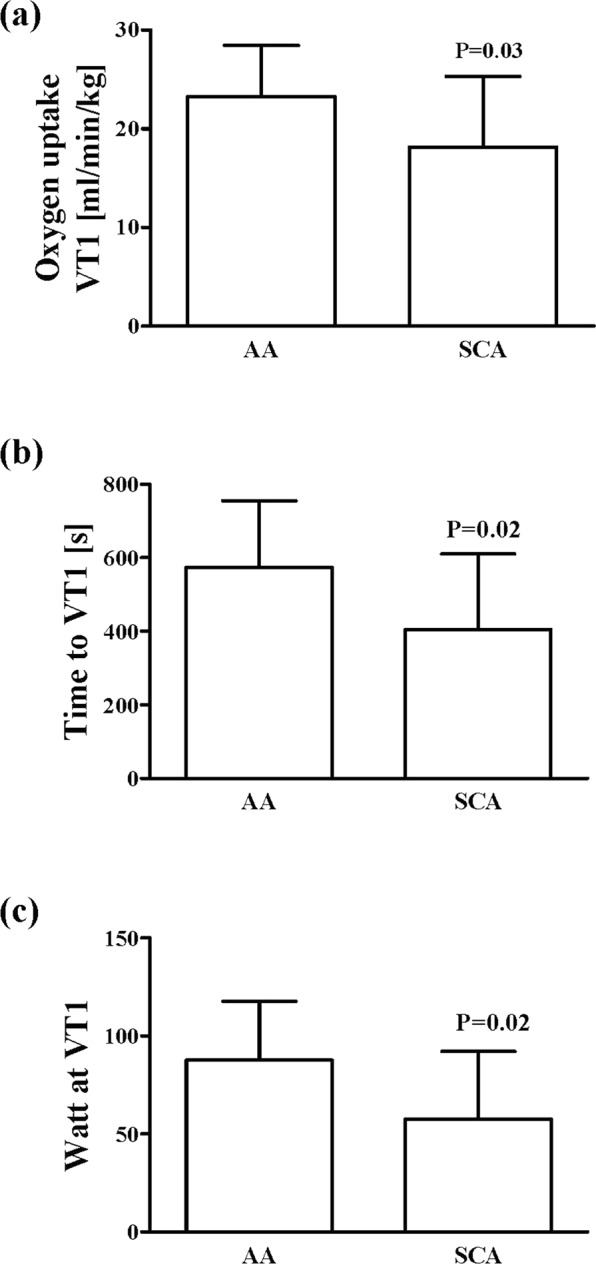
Reduced performance capacity in patients with sickle cell anemia. (**a**) Maximal oxygen uptake, (**b**) time to first ventilatory threshold (VT 1) and (**c**) watt power at VT 1 were significantly reduced in sickle cell patients (SCA) compared to healthy controls (AA). Data are mean ± s.d. of n = 8 AA and n = 8 SCA.

Results indicated a significant difference in VT 1 between AA and SCA (P = 0.03; Fig. 1a), as well as lower time to reach VT 1 (P = 0.02; Fig. 1b) and lower power at VT 1 (P = 0.02; Fig. 1c) in SCA. Lactate concentration measured at VT 1 was 2.5 mmol for SCA and 3.1 mmol for AA (P = 0.17).

### Exercise does not affect blood count and plasma free haemoglobin concentration in SCA

**Figure 2:**
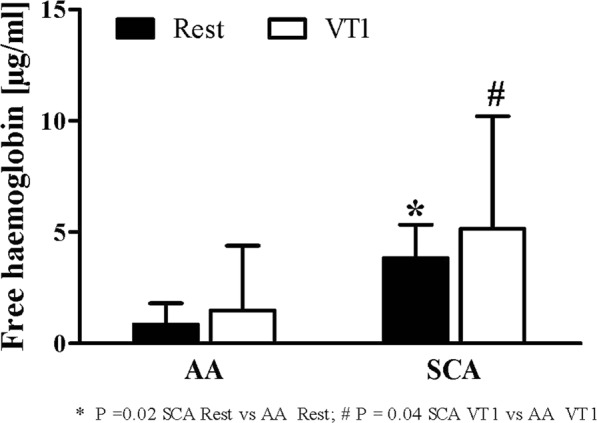
Exercise did not affect free haemoglobin concentration. SCA patients showed significantly higher free hemoglobin levels compared to healthy controls (AA). Exercise did not alter free hemoglobin levels in both SCA and AA. Data are mean ± s.d. of n = 8 AA and n = 8 SCA.

Data presented in Table 1 revealed significant difference in blood count between AA and SCA but no effect of exercise was observed in the two groups. Plasma free haemoglobin concentration was significantly higher in SCA compared to AA (P = 0.02 at rest and P = 0.04 at VT1) but was not significantly affected by the intervention (Fig. 2).

**Table 1:** Blood parameters in healthy controls (AA, n = 8) and sickle cell anaemia patients (SCA, n = 8) measured at rest and at VT 1.

	AA		SCA	
	Rest	VT 1	Rest	VT1
WBC [*10^3^/µl]	7.3 ± 2.3	6.9 ± 2.5	10.5 ± 2.7*^P=0.03^	9.9 ± 3.3^#P=0.03^
RBC [*10^6^/µl]	5.5 ± 0.9	5.3 ± 0.9	3.1 ± 1.4^*P=0.001^	2.7 ± 1.3^#P=0.0002^
PLT [*10^3^/µl]	199.9 ± 82.2	210.6 ± 104.8	88.9 ± 58.6	175.9 ± 194.8
Hb [g/dl]	15.2 ± 1.5	15.3 ± 1.3	10.3 ± 2.8^*P=0.0007^	9.7 ± 2.4^#P=0.00003^
Hct [%]	46.4 ± 5.9	44.5 ± 7.5	27.0 ± 7.7^*P=0.0006^	24.1 ± 8.4^#P=0.00007^
MCV [fl]	84.7 ± 5.5	84.3 ± 5.8	91.3 ± 12.8	91.6 ± 12.9
MCH [pg]	27.9 ± 2.6	29.6 ± 4.8	35.5 ± 9.0^*P=0.02^	38.3 ± 8.9^#P=0.01^
MCHC [g/dl]	32.9 ± 3.1	33.5 ± 9.5	38.4 ± 4.4^*P=0.02^	41.4 ± 6.2^#P=0.03^

Data are mean ± s.d. *SCA Rest vs AA Rest; ^#^SCA VT1 vs AA VT 1.

### RBC deformability is lower in SCA but is not affected by exercise

**Figure 3:**
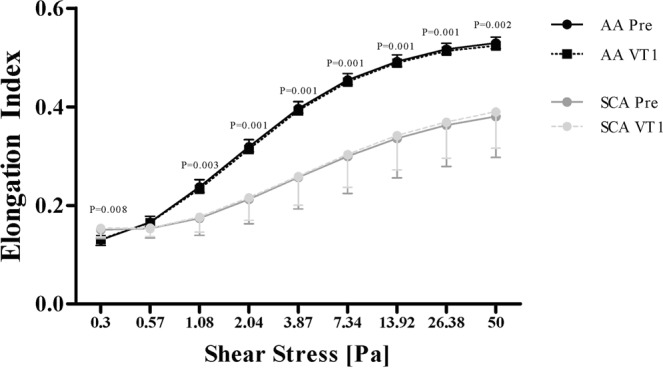
Exercise did not alter RBC deformability. RBC deformability was significantly lower in SCA patients at rest and VT1. Exercise did not affect RBC deformability in the tested groups. Data are mean ± s.d. of n = 8 AA and n = 8 SCA.

RBC deformability was significantly higher in AA for shear stresses between 1.08 and 50 Pa (1.08 Pa: P = 0.0003; 2.04–26.38 Pa: P = 0.0001; 50 Pa: P = 0.0002) while values were significantly lower in AA for a shear stress of 0.3 Pa (P = 0.008) when compared to SCA. Acute exercise did not affect RBC deformability (Fig. 3).

### Exercise decreases RBC NO production and RBC-NOS activation

**Figure 4:**
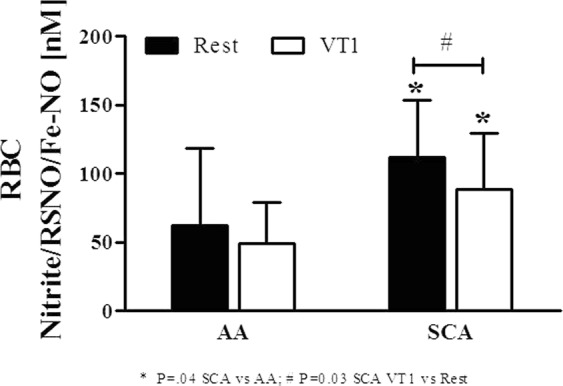
Exercise reduced RBC Nitrite/RSNO/Fe-NO concentration in SCA patients. RBC Nitrite/RSNO/Fe-NO levels were significantly higher in SCA compared to healthy controls (AA) at Rest. Significantly decreased Nitrite/RSNO/Fe-NO concentrations were measured in SCA after exercise (VT 1) while levels in AA remained unaffected. Data are mean ± s.d. of n = 8 AA and n = 8 SCA.

RBC Nitrite/RSNO/Fe-NO concentration was significantly higher in SCA compared to AA (P = 0.04 at rest and P = 0.04 at VT1). The acute exercise bout significantly reduced Nitrite/RSNO/Fe-NO in SCA (P = 0.03) while no effect was observed in AA (Fig. 4).

**Figure 5:**
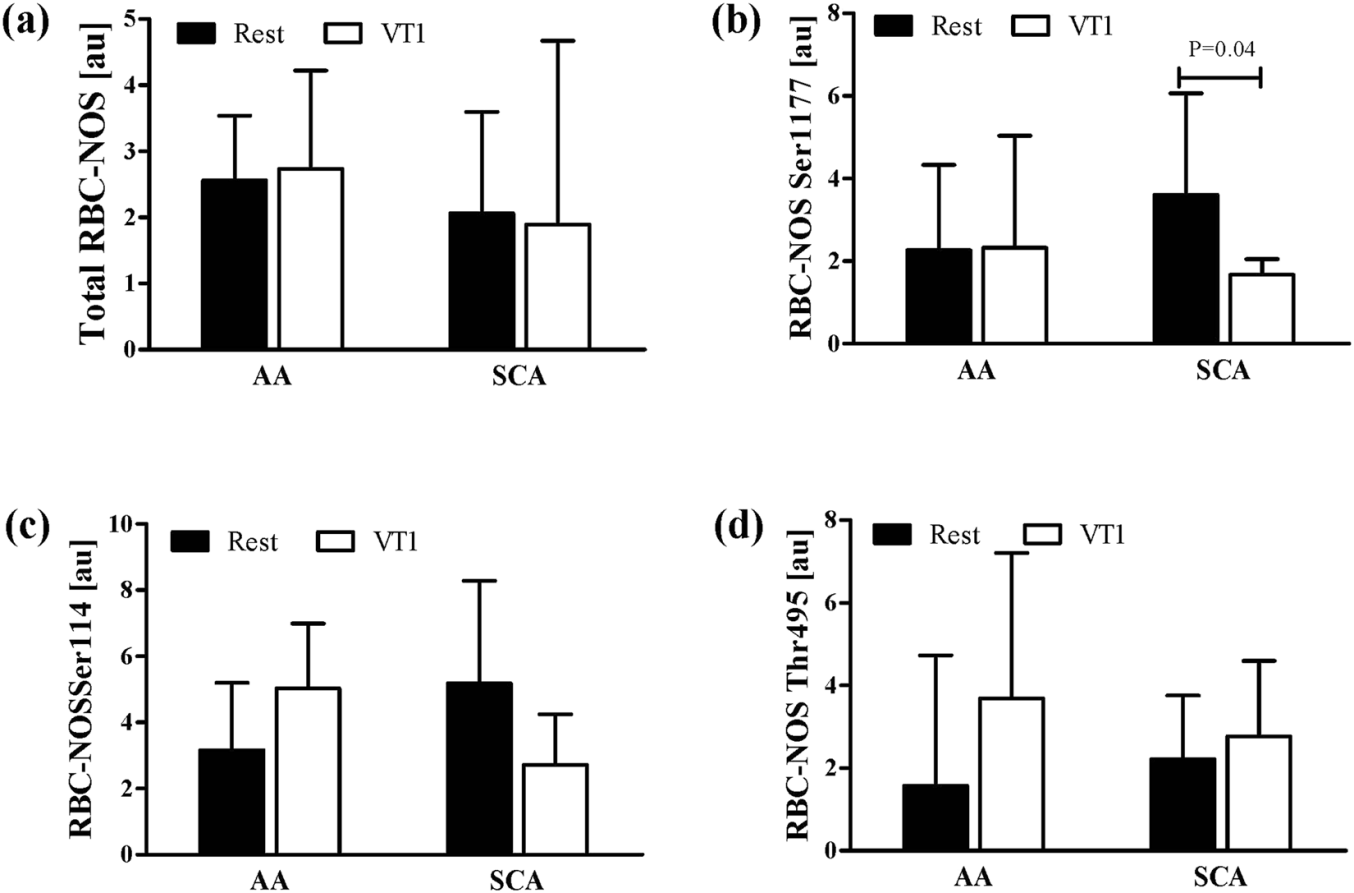
Exercise reduced activation of RBC-NOS enzyme in SCA. (**a**) Total RBC-NOS levels were comparable between the tested groups and not affected by exercise. (**b**) RBC-NOS serine 1177 staining was also comparable between SCA individuals and healthy controls (AA) at Rest but decreased at VT 1 in SCA patients. (**c**) RBC-NOS serine 114 and (**d**) RBC-NOS threonine 495 staining did not show differences between the tested groups and were unaffected by exercise. Data are mean ± s.d. of n = 5 AA and n = 6 SCA.

Immunohistochemical staining of total RBC-NOS showed no differences between AA and SCA and no effect of exercise on total RBC-NOS (Fig. 5a). Phosphorylation of RBC-NOS at serine 1177 residue showed no difference between AA and SCA at rest but phosphorylation significantly decreased at VT 1 in SCA (P = 0.4; Fig. 5b). RBC-NOS phosphorylation of the inhibitory phosphorylation residues serine 114 and threonine 495 were not significantly different between AA and SCA and were not affected by exercise (Fig. 5c,d).

### RBC oxidative stress is higher in SCA but is not affected by exercise

**Figure 6:**
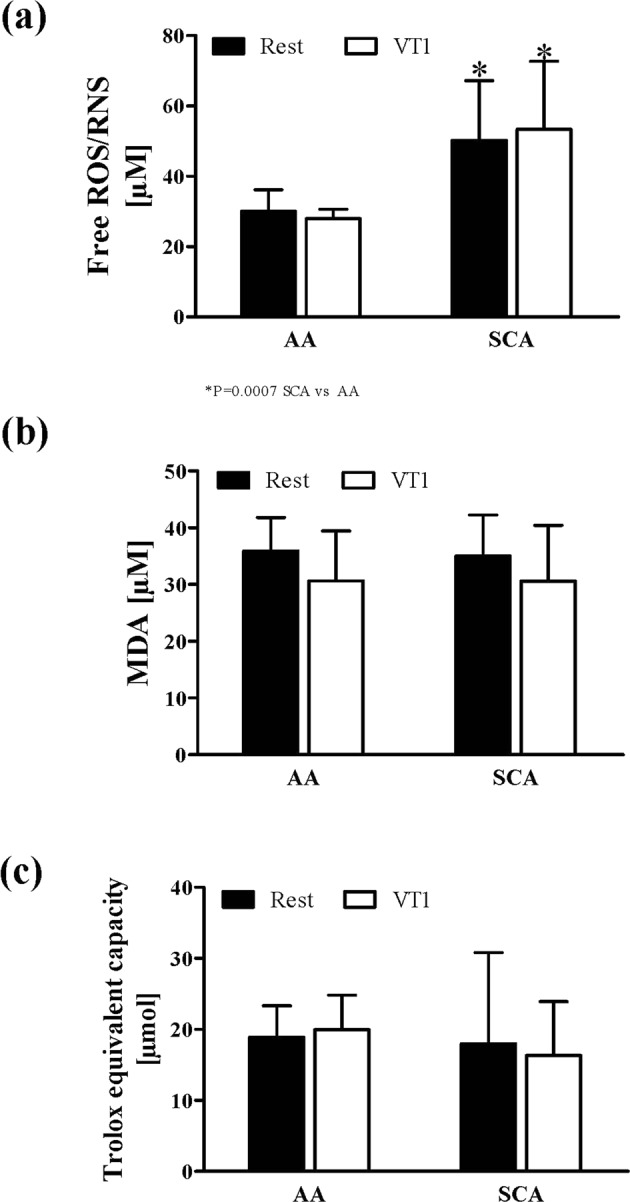
Exercise did not affect oxidative status in AA and SCA individuals. (**a**) Free ROS/RNS levels were significantly higher in SCA compared to healthy (AA) subjects but exercise did not affect free ROS/RNS levels. (**b**) Lipid peroxidation, represented by MDA concentration, and (**c**) total antioxidant capacity, represented by trolox equivalent capacity, were comparable between the groups and not affected by exercise. Data are mean ± s.d. of n = 8 AA and n = 8 SCA.

Free ROS/RNS level was higher in SCA compared to AA (P = 0.0007 at rest and at VT1, respectively) but was not affected by exercise (Fig. 6a). RBC malondialdehyde (MDA) levels were similar in AA and SCA and values were not affected by the acute exercise bout (Fig. 6b). Total antioxidant capacity, reflected by Trolox equivalent capacity, showed similar data in AA and SCA and was not affected by the exercise intervention (Fig. 6c).

## Discussion

This is the first study describing the effects of acute submaximal exercise on RBC deformability and RBC NO signalling in SCA patients. Key findings of the study indicate that an acute bout of submaximal exercise has no deleterious effects on RBC deformability and oxidative state of SCA patients but does reduce RBC-NOS activation and related NO production.

Physical fitness was reduced in the tested SCA adolescents compared to the healthy control group. Several authors report delayed growth and reduced physical fitness in SCA individuals compared to healthy control subjects because of chronic haemolytic anaemia described for SCA^29,31,32^. Reduced fitness might be also be associated to deficits in body composition such as reduced muscle mass and strength^33,34^. A study by Callahan^35^ further revealed that SCA patients show reduced peak oxygen uptake, low anaerobic threshold, gas exchange abnormalities and high ventilatory reserve. Abnormal cardiac function, mechanical ventilation limitation, pulmonary vascular disease, peripheral vascular impairment and muscle dysfunction are also involved in the reduction of physical fitness in SCA (see for review^27^).

As previously reported^5,17,36,37^, our results showed a decrease of RBC deformability and higher plasma free haemoglobin level in SCA compared to AA at rest. Plasma free haemoglobin values reported herein are lower compared to previously reported values^38^, possibly because of concentration losses during freezing/thawing process. Despite this limitation - which affected both SCA and AA samples - values were higher in SCA compared to AA, as previously reported. The chronic intravascular haemolysis is at the origin of the increase in free haemoglobin in plasma in SCA, which interferes with circulating NO to produce inert nitrate and decrease NO bio-availability. The decrease in NO bio-availability would contribute to SCA pathophysiology because it might promote vascular dysfunction, proliferative vasculopathy or other clinical complications^39^. The recent data indicate no further increase in plasma free haemoglobin during acute exercise which might indicate that the applied exercise protocol does not further promote intravascular haemolysis.

The reduction of RBC deformability in SCA increases fragility of RBC that become more prone to haemolysis compared to RBC of AA individuals^40^. RBC deformability in SCA is highly dependent on the presence of HbS polymers inside RBCs and the degree of RBC dehydration, which depends on the activation of the Gardos Channel and K^+^-Cl^−^ co-transporter^41^. Increased oxidative stress has also been shown to affect RBC rheology and fragility in SCA^42^. Indeed, free ROS/RNS levels were significantly higher in SCA compared to AA. However, exercise did not further alter RBC deformability and did not further increase free ROS/RNS levels in SCA. RBC deformability at rest measured at 0.3 Pa showed higher values in SCA compared to AA but this difference was not caused by the intervention. Instead, this finding might be explained by atypical responses upon shearing, indicating dysfunctional RBC membrane properties that might affect the laser diffraction pattern. Altered blood profile has already been reported for SCA^43,44^ and the results of the present study are thus in line with previous findings. The exercise intensity used in this study (i.e., VT1) caused only very slight changes in blood lactate concentration (around 2–2.5 mM), which does not significantly affect blood pH. In addition, there was no change in haemoglobin oxygen saturation during intervention (data not shown), which would suggest that this exercise is probably safe. We observed no change in MCH, MCHC, MCV or RBC deformability measured at nine shear stresses induced by exercise in SCA, as well in AA individuals. Although direct measurements of RBC sickling was not possible in this study, these results suggest that the applied exercise stimulus was not too intense and did not promote RBC sickling or cause further RBC alterations in SCA. Nevertheless, one may recognize that although not statistically significant, MCHC slightly increased from rest to VT 1 which could indicate that slight dehydration occurred. In addition, although measurements were done quickly after blood sampling, they might not completely reflect what happened in the body when patients were exercising. It seems likely that higher exercise intensity than in the present study could promote massive RBC sickling and could conduct to a decrease in RBC deformability.

RBC function may also be affected by NO bio-availability, which in RBC is produced by RBC-NOS^8^. Total RBC-NOS content and phosphorylation of the inhibitory residues serine 114 and threonine 495 were comparable between AA and SCA as previously shown^17^ and exercise did not alter staining intensities. RBC-NOS activation, reflected by serine 1177 phosphorylation, was similar in AA and SCA. It has been previously shown that RBC-NOS activation was higher in SCA who were not under hydroxycarbamide medication^17^. However, a recent study by Nader and collagues^45^ reported lower RBC-NOS serine 1177 phosphorylation in hydroxyurea treated than in non-treated SCA while no significant difference in RBC-NOS activation was detected between AA and SCA without hydroxyurea. The lack of difference in RBC-NOS activation between the two groups in the present investigation may be explained by the fact that some of the tested SCA patients received hydroxycarbamide medication while some did not but the analysed sample size is too small to divide the SCA group into hydroxycarbamide treated and non-treated. Though, exercise reduced RBC-NOS serine 1177 phosphorylation in SCA but not in AA, and this had an immediate effect on NO production because RBC Nitrite/RSNO/Fe-NO concentration decreased after exercise in SCA, although the concentration remained higher than in AA. This decrease in Nitrite/RSNO/Fe-NO levels observed in SCA after exercise is not explained by changes in oxidative state because associated parameters remained unaltered. In AA, reduction of RBC-NOS dependent NO production would decrease RBC deformability^9^. However, a previous study from our group^17^ showed that *in-vitro* high shearing of SCA RBCs (at it would occur during exercise) increased RBC-NOS activation but had no immediate effect on RBC deformability. It seems that the associations between RBC-NOS, NO and RBC deformability are more complicated in SCA than in AA. It has been reported that HbS would reduce nitrite a little bit faster than normal adult haemoglobin when it is not polymerized but polymerized HbS would reduce nitrite very slowly^46^. It is difficult to know how much these variations would account for the differences observed in this study. Further studies are needed to investigate the cellular mechanisms leading to a reduction in NO signalling in SCA, with or without hydroxycarbamide medication, during exercise.

Despite the reduction in RBC Nitrite/RSNO/Fe-NO content at the end of exercise in SCA, RBC deformability remained unaffected. RBC-NO derived production caused by exercise has been demonstrated to positively affect RBC deformability in healthy individuals^16^, probably through its effects on RBC cytoskeleton proteins by S-nitrosylation^9^. However, the submaximal exercise used in this study was unable to promote RBC-NOS activation and RBC NO production in AA, which suggests that exercise intensity and/or duration was probably too low to receive beneficial effects on RBC physiology, as well as on RBC rheology, in both AA and SCA individuals.

In conclusion, our results demonstrate that a short and acute submaximal exercise bout has no deleterious effects on RBC deformability or oxidative stress markers in SCA. However, the exercise stress stimulus used in this study could also be too low to provide beneficial RBC adaptations. Further studies are needed to test the effects of long-term training on RBC physiology and clinical status in SCA.

## Methods

### Study protocol

The protocols used in this study were reviewed and approved by the ethics committee of the German Sport University Cologne (date of approval 16.07.2014). The protocols are in line with the Declaration of Helsinki and all participants or legal guardians of the participants gave written informed consent to participate in this study.

A total of 16 adolescents were included in this study of which n = 8 were patients with SCA (HbF = 13.4 ± 7.9%, HbS = 84.1 ± 7.2%) and n = 8 were healthy controls (AA). Anthropometric data are shown in Table 2. SCA diagnosis was made by haemoglobin electrophoresis and confirmed by genetic studies in case of unclear electrophoresis results. Patients were at steady-state (i.e., without any acute vaso-occlusions or hospitalized complications and no blood transfusion in the last three months prior to intervention). Five out of eight patients received hydroxycarbamide medication (20–33 mg/kg body weight).

**Table 2:** Anthropometric data of healthy control (AA) and sickle cell anaemia patients (SCA).

	Gender	Age [years]	Height [m]	Weight [kg]
AA	3 f/5 m	19.1 ± 1.6	1.71 ± 0.1	63.25 ± 12.0*
SCA	4 f/4 m	17.4 ± 3.6	1.66 ± 0.1	53.00 ± 6.5

Data are mean ± s.d. *P = 0.03 SCA vs AA.

### Blood sampling

Venous blood samples were taken from the elbow vein pre (Rest) and post (VT 1) exercise test and anticoagulated using sodium heparin (BD; USA) or EDTA (BD; USA). EDTA blood was directly stored at −80 °C for measurement of the different haemoglobin fractions using capillary electrophoresis (Capillarys 2 Flex Piercing, Serbia) and sodium heparin blood was separated by centrifugation at 3600 g for 2 min and 4 °C. Aliquots of plasma supernatant were snap frozen and stored at −80 °C. RBC pellet was processed as described for the different parameters, snap frozen and stored at appropriate temperature until measurement.

### Performance parameters

A progressive submaximal bout of exercise was conducted on a cycle ergometer (ergoselect 150, ergoline, Germany) with breath-by-breath gas exchange analysis (Metalyzer 3B, Cortex Biophysics GmbH, Germany). The measurement started with a 2 min breath-by-breath measurement at rest. Initial workload was 20 Watt and workload increased every 2 min by 20 Watt. A cadence of 60 revolutions per minute was maintained. The measurement was stopped when the subject reached the first ventilatory threshold (VT 1) which defines the switch from aerobic to aerobic-anaerobic metabolism. Presented physical performance parameters include VT 1 [ml/min/kg], time to VT 1 [s] and power at VT 1 [W].

### Lactate

Whole blood was mixed with a haemolytic liquid using prepared tubes from EKF Diagnostics. Lactate concentration of the 1:50 dilution was directly analysed using the EKF-Biosen S-Line Lab + lactate analyser (EKF Diagnostics GmbH, Germany).

### Blood count

Basal blood parameters including red blood cells (RBC), white blood cells (WBC) and platelets (PLT) counts, haematocrit (Hct), haemoglobin concentration (Hb), mean corpuscular volume (MCV), mean corpuscular haemoglobin (MCH) and mean corpuscular haemoglobin concentration (MCHC) were directly determined in whole blood using the haematology analyser Sysmex Digitana KX-21N (Sysmex, Switzerland).

### Plasma free haemoglobin concentration

Plasma samples were thawed on ice and the Human Hemoglobin ELISA kit (Abcam, Cambridge UK) was applied to measure the free human haemoglobin concentration according to the instructions supplied by kit booklet.

### RBC deformability

Sodium heparin anticoagulated whole blood was mixed with a polyvinylpyrrolidone solution in a 1:250 ratio (PVP; 28 cP, RR Mechatronics, The Netherlands). RBC deformability was measured by a laser diffraction ektacytometer (Laser assisted optical rotational cell analyzer; LORCA; RR Mechatronics, The Netherlands). The system has been previously described^47^. Briefly, the blood/PVP solution was exposed to nine consecutive shear stresses, from 0.3 to 50 Pa. A laser beam was directed through the sample and the resulting diffraction pattern was analysed by the computer software. An elongation index (EI) was calculated at each shear stress using the long and short axes of the ellipse from the diffraction pattern (a and b, respectively) using the equation (1).

1 $${\rm{EI}}=({\rm{a}}-{\rm{b}})/({\rm{a}}+{\rm{b}}).$$

### RBC Nitrite/RSNO/Fe-NO

After separation of whole blood, RBC pellet was mixed with a nitrite preservation solution as described elsewhere^47^. Samples were thoroughly mixed, snap frozen and stored at −80 °C until measurement.

RBC Nitrite/RSNO/Fe-NO concentrations were measured according to published protocols^48,49^. Briefly, thawed RBC samples were mixed with ice-cold methanol (VWR International, Germany), centrifuged and the Nitrite/RSNO/Fe-NO levels of the supernatant were measured as described earlier^50^. Samples were injected into an acidified tri-iodide solution that reduces nitrite but also iron-nitrosylheme, and S-nitrosothiols to NO gas which was analysed by chemiluminescent reaction with ozone using a chemiluminescence NO detector (CLD 88e, EcoPhysics, Switzerland). All samples were measured in triplicate. The Chart FIA software (Ecophysics, Switzerland) was used to integrate the area under the curve and the sample Nitrite/RSNO/Fe-NO concentration was calculated by the use of standard solutions. RBC Nitrite/RSNO/Fe-NO concentration of the samples was corrected for Nitrite/RSNO/Fe-NO concentrations of methanol and preservation solution, respectively.

### Total RBC-NOS and activation state of RBC-NOS

For immunohistochemical staining of RBC proteins and protein activation state, RBC pellet was fixed in 4% formaldehyde as described earlier^16^. Blood smears were prepared and heat fixed. A test and a control area were marked on each slide using a grease pen. Both areas were washed with tris-buffered saline (0.1 mol TBS, pH 7.6) and incubated with 0.1% trypsin solution. RBC were then treated with a solution containing 2% hydrogen peroxide, 80% methanol, rest aqua dest and non-specific binding was minimized by incubation with 3% skim milk. The test area of each slide was incubated with the respective primary antibody in a 0.3% skim milk solution. Primary antibodies used for the analyses were as follows: Anti-phospho-eNOS (Thr495) (dilution: 1:400, Cell Signaling, USA), Anti-phospho-eNOS/NOS III (Ser114) (dilution: 1:500, Cell Signaling), Anti-phospho eNOS (Ser1177) (dilution: 1:150, Millipore, USA) and Anti-eNOS/NOS Type III (dilution: 1:700, BD, USA). Slides were washed with TBS, treated with 3% normal goat serum (Dako, Denmark) and incubated with a secondary goat anti-rabbit antibody (dilution: 1:400, Dako, Denmark). 3,3-diaminobenzidine-tetrahydrochloride (DAB) solution (Sigma, USA) in TBS was added to the slides and staining was stopped by adding TBS to the DAB solution. Slides were then dehydrated by exposure to alcohol solutions of increasing concentration and sealed using Entellan® (Merck, Darmstadt, Germany). Images were taken from the stained slides using a Zeiss microscope coupled to a CCD-camera (DXC-1850P, Sony, Germany). The semi-quantitative analysis of the grey values was conducted using the ‘Image J’ software (National Institutes of Health, USA). Grey values of a total of 50 RBC from at least four images were determined in the test area and subtracted from the background value, which was measured in a cell-free area of the slide in order to obtain staining intensity caused by the binding of the antibodies. Then, the grey values of a total of 10 RBC from at least two images were determined in the control area and also subtracted from the background value to obtain the baseline grey values of unstained RBC. Finally, grey values of RBC from test and control areas were subtracted to obtain net staining intensities.

### Oxidative status of RBC

#### RBC ROS

After separation of whole blood, RBC pellet was mixed with 0.1 mol phosphate buffered saline (PBS; pH 7.4) to obtain a 2 * 10^7^ cells/ml suspension, snap frozen and stored at −80 °C until measurements. Free ROS/RNS were measured using the OxiSelect *In Vitro* ROS/RNS Assay Kit (Cell Biolabs Inc., USA).

#### Lipid peroxidation

After separation of whole blood, RBC were diluted with 0.1 mol PBS (pH 7.4) to obtain 5 * 10^7^ RBC/ml, snap frozen and stored at −80 °C. Measurement of lipid peroxidation was performed by using the TBARS Assay Kit (Cayman Chemical, USA) according to the protocol supplied by the kit booklet.

#### Total antioxidant capacity

RBC pellet was transferred to a clean tube with 1 * 10^7^ RBC/ml in 0.1 mol PBS (pH 7.4), snap frozen and stored at −80 °C until measurement. Samples were thawed on ice and the total antioxidant capacity assay kit (Abcam, UK) was applied to measure RBC total antioxidant capacity according to the manufacturers´ instructions.

### Statistics

Statistical analyses and presentation of data were conducted using commercial software (Prism, GraphPad Software Inc., USA). All data are presented as mean ± standard deviation (s.d). Normal distribution of the data was tested using D´Agostino and Pearson omnibus normality test. Performance parameters were compared between AA and SCA using Mann-Whitney test with a confidence interval of 95%. A two-way ANOVA with repeated measures and Bonferroni post hoc test was used to determine group (AA and SCA), time (Rest and VT 1) and interaction effects.

## Data Availability

The data that support the findings of this study are available on reasonable request from the corresponding author M.G. The data are not publicly available due to restrictions described in the signed patient information letter ensuring that individual data will not be presented publicly in order to ensure the privacy of research participants.
